# Activity Metrics from the ActiGraph GT9X Link and ActiGraph LEAP Using an Open-Source R Package

**DOI:** 10.53520/rhm2026.104225

**Published:** 2026-09-21

**Authors:** Jethro Raphael M. Suarez, Chitra Banarjee, Kworweinski Lafontant, Rui Xie, Ladda Thiamwong

**Affiliations:** 1 College of Nursing, University of Central Florida, Orlando, FL, USA; 2 College of Medicine, University of Central Florida, Orlando, FL, USA; 3 Institute of Exercise Physiology and Rehabilitation Science, College of Health Professions and Sciences, University of Central Florida, Orlando, FL, USA; 4 Department of Statistics and Data Science, College of Sciences, University of Central Florida, Orlando, FL, USA; 5 Disability, Aging, and Technology Cluster, University of Central Florida, Orlando, FL, USA

**Keywords:** ENMO, accelerometer, movement, wearable sensor

## Abstract

**Introduction:**

Accelerometers are a common method of direct activity measurement, but the continuous release of newly developed accelerometer device models warrant comparison with previously validated models. The performance of the ActiGraph LEAP, the successor to the widely used ActiGraph GT9X Link, has rarely been compared directly with other accelerometer models.

**Methods:**

Healthy young adults wore both the ActiGraph LEAP and GT9X Link on their non-dominant wrist for seven consecutive days in free-living conditions. An open-source R package (GGIR) was utilized to quantify activity from raw accelerometer data. Raw accelerometer data files were resampled to a common sample rate (30 Hz) and timestamp matched by minute. Aggregated activity metrics, specifically average daily acceleration and peak 30-minute acceleration, were compared through intraclass correlation coefficients (ICC_2,1_), mean absolute percent error (MAPE), and equivalence tests utilizing ±10% equivalence bounds. Bland-Altman plots visualized agreement.

**Results:**

A total of 25 young adults were included (21.2 ± 4.0 years) with an average of 4.1 days of valid wear (≥ 10 hours). Both activity metrics exhibited good to excellent agreement, low MAPE values (<10%), and passed equivalency tests. Additionally, significant proportional bias was not found when analyzing the Bland-Altman plots for each activity metric.

**Conclusions:**

The performance of the ActiGraph LEAP is comparable to that of the ActiGraph GT9X Link among healthy young adults, as seen through comparison of aggregated acceleration metrics quantified using the GGIR method.

## Introduction

Wearable devices, specifically accelerometers, are one of the most common methods of direct activity measurement among individuals in free-living conditions. Numerous brands of accelerometers have been used for activity measurement, including the activPAL, Axivity AX3, and GENEActiv devices^1–3^. However, accelerometers are continuously being improved, with new accelerometer models being released with little knowledge of their performance in comparison to previously validated models. A notable example of this lies within one of the leading accelerometer brands, ActiGraph LLC (rebranded as Ametris as of 2025). Numerous accelerometers from the ActiGraph company have been used for activity measurements among a wide variety of populations^4–6^. Specifically, the ActiGraph GT9X Link has been a widely used accelerometer for activity measurement^7–9^. However, the GT9X Link was discontinued by ActiGraph as of August 2023. Shortly after this discontinuation, ActiGraph released the ActiGraph LEAP, referred to by the company as the successor to the GT9X Link, in November of 2023. While considered to be the successor, knowledge of the performance of the LEAP device in comparison to its predecessor, as well as any other accelerometer, is scarce. Previous work from our lab group directly compared the performance of the ActiGraph LEAP with the GT9X Link in physical activity (PA) measurement through Monitor Independent Movement Summary (MIMS) units and found excellent agreement in recorded sedentary behavior, light PA, and moderate-to-vigorous PA, as seen through intraclass correlation coefficient (ICC) values above 0.90^10^. The MIMS unit was utilized to reduce variability between devices, as the minimum difference in sampling rates that can be achieved between the two devices is 2 Hz. However, quantifying activity through MIMS units remains a less widely adopted accelerometer processing method than other accelerometer processing methods, thereby limiting the generalizability of these findings.

Activity counts are a commonly used acceleration metric for quantifying PA levels among ActiGraph devices^11,12^. However, activity counts are specific to Actigraph devices and therefore are not generalizable across other accelerometer devices. An open-source package in R software, referred to as GGIR, has been widely used for quantifying aggregated activity from raw accelerometer data^13^. GGIR provides established activity metrics that allow for comparisons across studies. Establishing whether GGIR-derived metrics produce comparable estimates between the ActiGraph LEAP and GT9X Link supports the findings of the previous comparative work. The aim of this study was to use the GGIR method to quantify aggregated activity metrics, specifically average daily acceleration and average daily 30-minute peak acceleration, from raw accelerometer data produced by the ActiGraph GT9X Link and ActiGraph LEAP and directly compare the calculated values to determine their level of agreement. Based on previous work, it is hypothesized that the measurements from the two devices will be comparable, as found by ICC values higher than 0.90, low mean absolute percent error (MAPE), passing equivalency testing, and no evidence of significant proportional bias.

## Methods

This study was a secondary analysis of data from a previously published study that took place in the Greater Orlando, Florida area^10^. Recruitment was on a rolling basis and accomplished through word-of-mouth, physical flyers posted across public community boards, and virtual advertisements through a university website. Recruitment and implementation of all study procedures were conducted between December 2024 and April 2025.

### Participants

The inclusion criteria for this study required that participants were 18 years of age or older, spoke and understood English, and resided in the Central Florida area. Participants were excluded if any open wounds were present near or at the wrist and forearm area or if they could not stay in the Central Florida area for a consecutive seven-day period. Interested participants were screened using an electronic survey. If participants passed the screening requirements, electronic consent was obtained through a virtual consent form, followed by a demographic survey that included items such as self-reported height, weight, and race/ethnicity. All surveys, as well as the survey responses, were stored within Research Electronic Data Capture (REDCap), a web-based application that is designed to securely store data for research purposes^14,15^. Participants who successfully completed all electronic forms were contacted by a member of the research team to schedule their initial in-person study session. The protocol for this study was reviewed and approved by the Institutional Review Board at the University of Central Florida (STUDY00007331) and adhered to the Declaration of Helsinki.

### Protocol

A total of two in-person visits occurred for all participants. At the initial visit, a member of the research team first reviewed the consent form with the participant to ensure comprehension of the study procedures. Following this review session, a researcher initialized an ActiGraph LEAP and ActiGraph GT9X Link (Ametris, Pensacola, FL, USA) simultaneously and placed both devices on the participant’s non-dominant wrist. Both devices were selected from pools of more than 30 respective units and multiple device pairs were used concurrently throughout the study duration. The GT9X Link was placed on the left side of the LEAP, despite being placed on either the left or right wrist. Out of the two devices, the LEAP is the only device to utilize an external button, specifically located on the right face of the device. It was found that a 20-second or more press of the button caused the device to reset, thereby halting data recording and erasing any recorded data. For this reason, the order of devices never changed to ensure that the external button was never pressed by the adjacent device (GT9X Link). Participants were instructed to continuously wear both devices on their nondominant wrist for seven consecutive days and only remove during bathing or to clean the devices. The second visit, approximately seven days following the initial visit, involved participants returning both devices and a researcher downloading data from each device immediately upon return. Participants were compensated with research credit that could be used for their university classes. All data was deidentified and stored in REDCap.

### Instrumentation and Activity Determination

The GT9X Link and LEAP devices share numerous characteristics, such as the use of a triaxial accelerometer with a dynamic range of ±8 gravitational units (*g*) and being water-resistant up to one meter. However, regarding measurement, the devices use differing software. For this study, ActiLife software (version 6.13.6, ActiGraph LLC) was used for device initialization and downloading of data for the GT9X Link, whereas ActiSync software (version 3.20.0, ActiGraph LLC) and the web-based program CentrePoint (ActiGraph LLC) was used for the LEAP. In addition, a common sampling rate is not shared between the devices. The GT9X Link exhibits eight different sampling rates ranging from 30 to 100 Hz in increments of 10 Hz, whereas the LEAP only allows for two sampling rates: 32 and 64 Hz. For the purposes of this study and to minimize the differences between the two devices, the sampling rate of the GT9X Link was set to 30 Hz, whereas the LEAP was set to 32 Hz.

Aggregated activity was determined from raw accelerometer data presented in .csv format from each device. An open-source R package that has been widely used for PA quantification, named GGIR, was utilized (version 3.3.4) in R software (version 4.4.0; Posit, Boston, MA, USA). GGIR revolves around the use of the Euclidean Norm Minus One (ENMO) metric for representing activity and overall movement^13^. The steps involved in GGIR processing include autocalibration of acceleration values to local gravity, detection of non-wear time (2023 method used for this study) and abnormally high acceleration values, and calculation of ENMO values (average magnitude of acceleration corrected for gravity)^13,16,17^. For ActiGraph devices, .gt3x files are commonly used for processing with GGIR, but the CentrePoint interface for the LEAP does not allow for the creation of .gt3x files at the time of this study. Due to this, a method of processing raw accelerometer data in .csv format using GGIR was needed. Previous work found that GGIR can read raw accelerometer data in .csv formats from the LEAP device^18^. However, the process has been optimized for data files produced from CentrePoint and the LEAP device. The original process suggested removing the timestamp column from the raw accelerometer data and solely relied on “start time” and “download time” that were explicitly defined in the .csv files. While this method reduced the overall .csv file sizes being read, the processing time was very slow due to the creation of timestamps during processing. To decrease processing time and increase efficiency, the timestamps were kept but reformatted to match the POSIXct date and time format, as GGIR seems to be most compatible with processing timestamp data in this format. The timestamp reformatting was only performed for .csv files from the LEAP devices, as ActiLife software is capable of outputting raw accelerometer data files with POSIXct timestamps from the GT9X Link devices. In addition to format conversion, raw LEAP files also underwent a resampling process using the “resample” function in R to transform the sampling rate from 32 Hz to 30 Hz, thereby ensuring comparability with the GT9X files. Prior to data processing through GGIR, timestamp matching between the files from each device for each participant was performed using the “lubridate” package in R after resampling, which involved reading raw accelerometer data recorded by each device for each participant, identifying unique minutes recorded by each device, matching shared minutes between devices, and removing all minutes not shared between both devices.

Many different variables can be calculated using GGIR, including sleep variables along with PA. However, for the purposes of this study, only average daily acceleration and average daily 30-minute peak acceleration were used for comparative analysis among days with a valid amount of wear time (≥ 600 minutes or 10 hours) since these continuous activity metrics provide measures of activity volume and intensity without relying on population-specific ENMO PA thresholds. Average daily acceleration was calculated as the sum of the mean daily acceleration values across all valid days of wear divided by the participant’s number of valid days of wear. Average daily 30-minute peak acceleration was calculated as the sum of the mean acceleration during the 30 most active minutes of each valid day of wear divided by the participant’s number of valid days of wear. Only valid days of wear that were shared by both devices were included in analysis. The file conversion, resampling, timestamp matching, and GGIR processing code have been included as supplementary material.

### Statistical Analysis

R software (version 4.4.0) was used for all statistical analyses. To determine agreement in aggregated activity metrics between devices, intraclass correlation coefficients (two-way random effects absolute agreement; ICC_2,1_) were determined and Bland-Altman plots were generated. Classification of ICC_2,1_ values were defined as: poor agreement (ICC_2,1_ < 0.50), moderate agreement (0.50 ≤ ICC_2,1_ < 0.75), good agreement (0.75 ≤ ICC_2,1_ < 0.90), and excellent agreement (ICC_2,1_ ≥ 0.90)^19^. Bland-Altman plots were overlayed with calculated 95% limits of agreement (LOA) and a mean difference line. Additionally, a linear regression line was included to determine if proportional bias existed between device measurements. T-tests determined if the slope of the regression lines significantly differed from zero, thereby indicating proportional bias. Mean absolute percent error (MAPE) was calculated with the aggregated activity metrics from the GT9X Link serving as the reference values and equivalency tests using the confidence interval method were performed^20^. Equivalence bounds were set as ±10% of the reference mean. These statistical tests follow closely with previous work that compared the performance of the presented devices, as well as among other devices^10,21–24^. Potential outliers in between-device differences for each aggregated activity metric were assessed using boxplots and defined as values lying more than 1.5 times the interquartile range. As a sensitivity analysis, all statistical tests were conducted twice under different conditions: 1) including all participants and 2) including only participants with at least four valid days of wear. A significance level of *p* < 0.05 represented statistical significance.

## Results

A total of 41 young adults were recruited for this study, but only 28 completed the study in its entirety after screening for inclusion/exclusion criteria. After removing participants with no valid days of wear, a total of 25 young adults were included in analysis (mean age = 21.2 ± 4.0 years). An average of 4.1 valid days of wear were exhibited by participants. The demographic characteristics of all included participants are presented in Table 1. The number of valid days calculated for each participant, as well as the calculated activity metrics for each device for each participant, are presented in Table 2.

No outliers were found for either metric. When including all participants regardless of the number of valid days of wear, ICC_2,1_ values greater than 0.90 were found for both activity metrics. Average daily acceleration and average daily 30-minute peak acceleration exhibited an ICC_2,1_ of 0.97. Average daily acceleration exhibited a slightly lower MAPE (6.71%) than that of average daily 30-minute peak acceleration (6.87%). Both activity metrics passed equivalency testing with equivalence bounds set at ±10% of the reference mean. Additionally, the slope of the regression line for each metric did not significantly deviate from zero (*p* > 0.05), suggesting that significant proportional bias was not present for either activity metric. When isolating participants with at least four valid days of wear, the findings were generally consistent with the results from the analysis that included all participants, with the exception of the ICC_2,1_ value for average daily peak 30-minute acceleration (0.84). However, analysis using 10,000 bootstrap replications found that the difference between the ICC_2,1_ values for average daily 30-minute peak acceleration was not statistically significant between groups. The results for all statistical analyses are presented in Tables 3 and 4. Bland-Altman plots overlayed with lines representing linear regression and the 95% LOA are presented in Figure 1, whereas the numerical values corresponding to each plot are presented in Table 5.

## Discussion

The purpose of this study was to directly compare the performance of the GT9X Link and LEAP devices in measuring aggregated activity metrics quantified using an open-source R package, GGIR. Specifically, average daily acceleration and 30-minute peak acceleration were compared. The original hypothesis was generally supported, as MAPE values were low (<10%), no significant proportional bias was found when examining Bland-Altman plots, and both metrics passed equivalency testing. While ICC_2,1_ values for both activity metrics were found to surpass the ICC_2,1_ threshold for excellent agreement (0.90) when examining all participants regardless of the number of valid days of wear, the ICC_2,1_ for average daily 30-minute peak acceleration fell below the excellent ICC_2,1_ threshold of 0.90 to 0.84 when including only participants that exhibited at least four valid days of wear. However, sensitivity analysis found that the difference between the ICC_2,1_ values was not statistically significant between groups. These results show that the performance of the LEAP device is comparable to that of the GT9X Link with respect to aggregated activity metrics quantified by the GGIR method.

As mentioned previously, prior work has compared the performance in measuring PA between the two devices presented in this study through MIMS units^10^. However, the thresholds for MIMS, specifically for common PA classification categories (SB, LPA, and MVPA), have yet to be validated. Despite previous studies relating MIMS PA thresholds to other known thresholds for widely used accelerometer units^25,26^, the utilization of the MIMS threshold for MVPA found an unrealistic amount of MVPA values in a sample of community-dwelling older adults. Average daily MVPA values were found to be in the 300-minute (5 hour) range, which did not seem plausible when compared to previously published norms^27,28^. It is possible that the MIMS threshold previously used may be too low for classifying MVPA. Validation studies comparing the suggested MIMS threshold against reliable measures of MVPA (e.g., metabolic equivalents via indirect calorimetry) would be needed to fully determine if the threshold is appropriate. For these reasons, a widely used method for quantifying activity (GGIR) was sought after to further compare the performance of the two devices being presented. Aggregated acceleration metrics were utilized as opposed to specific PA metrics (e.g., LPA and MVPA) since varying ENMO thresholds for PA categories exist in the literature for various populations and universally accepted thresholds have not yet been established^29–35^.

While the results of this study follow closely with the previous work^10^, differences were found during data processing procedures. From the previous comparison study, a total of 149 days of valid wear were compared between devices as opposed to the 103 valid days included in this study. This could primarily be attributed to the use of different non-wear algorithms. The previous validation study recreated an algorithm that classified non-wear by continuously scanning for periods with a continuous set of values below a suggested magnitude threshold^36^, whereas the GGIR package classified non-wear by continuously scanning for periods with values under a magnitude and standard deviation threshold^37^. The GGIR non-wear algorithm utilizes the variability and magnitude of acceleration values when determining non-wear periods, whereas the previously recreated algorithm focused primarily on magnitude. For this reason, the two approaches differ in sensitivity to prolonged periods of low movement. These differences in methodology likely contributed to the differences in valid days.

Equivalency testing and MAPE are commonly used when comparing the performance of devices. The previous comparison of the performance of the ActiGraph GT9X Link and LEAP utilized equivalence bounds of ±5% of the reference mean for increased statistical rigor^10^. Although the aggregated activity metrics examined in the current study did not satisfy this more rigorous threshold, they met equivalency within ±10% of the reference mean. Such equivalence bounds have been previously adopted based on industry standards and follow closely with previous accelerometer comparison studies^21–24^. A universally accepted threshold for MAPE when comparing acceleration metrics across accelerometer devices has not yet been established. However, the MAPE values found for both activity metrics within the current study are significantly lower than those found in previous literature that compared ENMO acceleration metrics across multiple accelerometers^38^. Additionally, another study assessing the validity of an activity monitor (Polar M430) through MAPE analysis deemed an MAPE value of 6.94% as acceptable^39^. As the MAPE values found within the present study are comparable to this value, it is suggested that the performance between the devices is comparable with respect to aggregated activity metrics.

It was found through Bland-Altman plots that the range of the 95% LOA for average daily acceleration was approximately 12 mg, regardless of group (Table 5). Additionally, the range of the 95% LOA for average daily 30-minute peak acceleration was approximately 54 mg (Table 5). Similar to MAPE, a universally accepted threshold for the range of the 95% LOA for Bland-Altman plots with respect to aggregated acceleration metrics currently has not been established and therefore, comparison to previous studies is beneficial. A previous study compared acceleration metrics between various wearable devices, with a mechanical shaker table serving as the reference, and stated that raw accelerometry data from Apple, Garmin, and Fitbit wearables can be considered reliable^24^. While these devices were not found to pass equivalency testing with ±10% of the reference, the ranges of the 95% LOA for the acceleration metrics analyzed in the present study were significantly less than the ranges for the devices being tested in the previously mentioned study^24^. These findings further support the idea that performance between the GT9X Link and LEAP devices is comparable.

Although the results show that the performance of the two devices is comparable, limitations for this study exist. Despite good to excellent agreement being found for both activity metrics between the devices, this study focused only on aggregated data. Aggregation of data may have reduced variability that occurred throughout the day. It is possible that acceleration measured by the devices differs at higher resolutions, such as at the minute-level. Previous work has found that accelerometers placed at the hip are more accurate when measuring PA in comparison to accelerometers placed at the wrist^40^. However, the wrist was chosen for device placement for this study due to expected higher compliance of wear in comparison to other locations. These results may not be generalizable to accelerometers placed at other locations on the body. Additionally, the placement of the devices on the wrist may have affected the results. Previous comparative studies have taped accelerometers on the wrist together, as opposed to placing them adjacently via individual watch bands^41^. Such setup may improve comparison between devices, but participants may not be willing to wear the devices if they are not properly secured. Although each device was selected from a pool of more than 30 respective units and multiple pairs of devices were used concurrently, systematic randomization of the units and documentation of individual unit serial numbers were not performed and therefore, unit-specific bias was not completely controlled for. Finally, this study only included healthy young adults due to recruitment methods primarily being conducted at a local university. The sample size comprising of solely young adults limits the generalizability of the findings. Future work should conduct comparative analysis on time-series data computed using the GGIR method, place the accelerometers on parts of the body other than the wrist, consider securely stacking accelerometer devices to ensure consistent movement between devices, document unit serial numbers prior to device deployment, perform systematic randomization when selecting devices from unit pools, and include a variety of age groups to increase the generalizability of the findings.

## Conclusions

Aggregated activity metrics, specifically average daily acceleration and 30-minute peak acceleration, from the ActiGraph GT9X Link and LEAP quantified using the open-source R package GGIR were compared, and good to excellent agreement between devices was found. These findings support previous work that found that the performance of the LEAP is comparable to the GT9X Link. Overall, aggregated activity metrics can be determined from the LEAP device using the proposed method: use the GGIR method to determine aggregated activity metrics from the LEAP after converting raw accelerometer timestamp data to POSIXct format. While the results of the present study show that the performance of the devices is comparable, these findings may be limited to healthy young adults, given the characteristics of the participants included in this study. Further comparison among varying age groups should be made. Additionally, if PA categorization is to be implemented when using the presented devices or the presented method, researchers and clinicians should carefully select ENMO PA thresholds based on the age of the population being assessed.

## Supplementary Material

**Supplementary Material.** The R codes utilized for this study can be found at https://osf.io/bhy3j/overview.

## Figures and Tables

**Figure 1.**
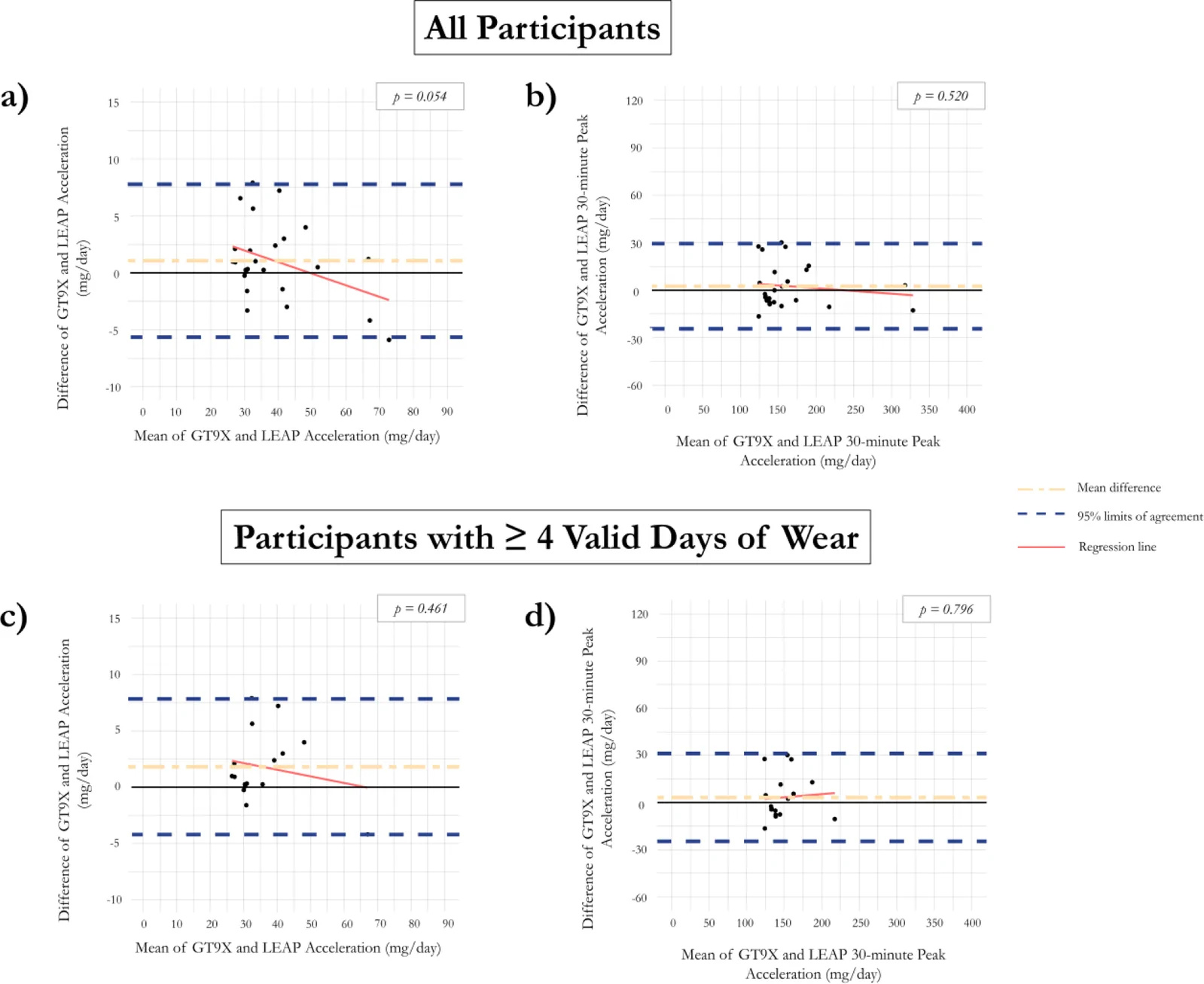
Bland-Altman plots for each aggregated activity metric for all participants (a and b) and participants with at least four days of valid wear (c and d). Significance of regression lines is represented by *p*-values shown within each plot.

**Table 1.** Participant demographics (N = 25).

Variable	Value

**Age** (years)	21.2 ± 4.0
**BMI** (kg/m^2^)	24.3 ± 4.0
**Sex**	
Female	19 (76%)
Male	6 (24%)
**Dominant hand**	
Left	3 (12%)
Right	22 (88%)
**Race/ethnicity**	
African American	3 (12%)
Asian	3 (12%)
Hispanic	2 (8%)
White	17 (68%)
**Self-rated overall health**	
Excellent	3 (12%)
Very Good	13 (52%)
Good	8 (32%)
Fair	1 (4%)
Poor	0 (0%)

Data are Means ± SD or n (%).

**Table 2.** Descriptive statistics for aggregated acceleration metrics across all included participants.

Participant	Days of Wear	GT9X		LEAP	
	
		Average Daily Acceleration (mg/day)	Average Daily 30-minute Peak Acceleration (mg/day)	Average Daily Acceleration (mg/day)	Average Daily 30-minute Peak Acceleration (mg/day)

1	6	28.26	140.59	26.15	148.17
2	6	30.50	134.24	30.24	143.07
3	2	33.77	131.40	32.75	137.80
4	6	29.99	115.87	31.58	132.37
5	5	43.18	156.26	40.18	153.84
6	5	43.91	169.31	36.69	139.12
7	6	40.31	165.14	37.92	159.59
8	2	32.05	141.75	25.51	116.00
9	5	36.37	137.64	28.46	109.94
10	1	40.53	145.00	41.96	145.01
11	6	35.79	131.19	35.54	134.97
12	4	31.10	131.13	30.78	133.58
13	5	30.50	130.60	30.43	134.96
14	2	51.91	198.10	51.42	182.66
15	6	32.63	150.93	30.68	139.45
16	6	35.35	173.22	29.71	145.77
17	6	29.92	135.52	30.16	140.69
18	5	27.74	134.58	26.81	142.44
19	5	27.00	127.66	26.00	123.06
20	1	29.17	149.31	32.48	159.30
21	1	41.05	170.40	44.04	176.70
22	1	69.79	322.00	75.68	334.70
23	2	67.24	319.40	66.04	316.30
24	5	50.07	193.83	46.07	180.91
25	4	64.98	212.14	69.16	222.64

**Table 3.** Averaged acceleration metrics across all participants and results from statistical analyses.

Variable	GT9X Link Mean (Reference)	LEAP Mean	Equivalence Bounds (± 10% of Reference Mean)	ICC_2,1_ (95% CI)	MAPE

**Average Daily Acceleration (mg/day)**	39.32 ± 12.51	38.26 ± 13.83	(−3.93, 3.93)	0.97 (0.93, 0.99)	6.71%
**Average Daily 30-minute Peak Acceleration (mg/day)**	164.69 ± 52.72	162.12 ± 54.56	(−16.47, 16. 47)	0.97 (0.93, 0.99)	6.87%

Data are Means ± SD, unless otherwise stated; CI: confidence interval; ICC_2,1_: intraclass correlation coefficient; MAPE: mean absolute percent error.

**Table 4.** Averaged acceleration metrics across participants with at least four valid days of wear and results from statistical analyses.

Variable	GT9X Link Mean (Reference)	LEAP Mean	Equivalence Bounds (±10% of Reference Mean)	ICC_2,1_ (95% CI)	MAPE

**Average Daily Acceleration (mg/day)**	36.33 ± 9.83	34.50 ± 10.52	(−3.63, 3.63)	0.95 (0.88, 0.98)	6.52%
**Average Daily 30-minute Peak Acceleration (mg/day)**	149.40 ± 25.79	146.15 ± 24.87	(−14.94, 14.94)	0.84 (0.62, 0.94)	7.38%

Data are Means ± SD, unless otherwise stated; CI: confidence interval; ICC_2,1_: intraclass correlation coefficient; MAPE: mean absolute percent error.

**Table 5.** Numerical characteristics of each Bland-Altman plot.

Variable	All Participants				Participants with ≥ 4 Valid Days of Wear			

	Mean Bias (95% CI)	95% LOA		95% CI	Mean Bias (95% CI)	95% LOA		95% CI

**Average Daily Acceleration (mg/day)**	1.07 (−0.35, 2.48)	UB:	7.78	(5.33, 10.23)	1.83 (0.25, 3.40)	UB:	7.85	(5.09, 10.60)
		LB:	−5.64	(−8.09, −3.20)		LB:	−4.20	(−6.95, −1.44)
**Average Daily 30-minute Peak Acceleration (mg/day)**	2.57 (−3.10, 8.23)	UB:	29.47	(19.66, 39.29)	3.25 (−4.08, 10.58)	UB:	31.20	(18.42, 43.99)
		LB:	−24.34	(−34.16, −14.53)		LB:	−24.70	(−37.49, −11.92)

CI: confidence interval; LB: lower bound; LOA: limits of agreement; UB: upper bound.
